# Accelerated Diffusion-Weighted MR Image Reconstruction Using Deep Neural Networks

**DOI:** 10.1007/s10278-022-00709-5

**Published:** 2022-11-04

**Authors:** Fariha Aamir, Ibtisam Aslam, Madiha Arshad, Hammad Omer

**Affiliations:** 1 Medical Image Processing Research Group (MIPRG), Electrical & Computer Engineering Department, COMSATS University Islamabad, Islamabad, Pakistan; 2 Service of Radiology, Faculty of Medicine, Geneva University Hospitals, University of Geneva, Geneva, Switzerland

**Keywords:** DWI, MRI, Deep learning, Single-shot echo planar imaging (ss-EPI), U-Net, Compressed Sensing (CS)

## Abstract

Under-sampling in diffusion-weighted imaging (DWI) decreases the scan time that helps to reduce off-resonance effects, geometric distortions, and susceptibility artifacts; however, it leads to under-sampling artifacts. In this paper, diffusion-weighted MR image (DWI-MR) reconstruction using deep learning (DWI U-Net) is proposed to recover artifact-free DW images from variable density highly under-sampled *k*-space data. Additionally, different optimizers, i.e., RMSProp, Adam, Adagrad, and Adadelta, have been investigated to choose the best optimizers for DWI U-Net. The reconstruction results are compared with the conventional Compressed Sensing (CS) reconstruction. The quality of the recovered images is assessed using mean artifact power (AP), mean root mean square error (RMSE), mean structural similarity index measure (SSIM), and mean apparent diffusion coefficient (ADC). The proposed method provides up to 61.1%, 60.0%, 30.4%, and 28.7% improvements in the mean AP value of the reconstructed images in our experiments with different optimizers, i.e., RMSProp, Adam, Adagrad, and Adadelta, respectively, as compared to the conventional CS at an acceleration factor of 6 (i.e., *AF* = 6). The results of DWI U-Net with the RMSProp, Adam, Adagrad, and Adadelta optimizers show 13.6%, 10.0%, 8.7%, and 8.74% improvements, respectively, in terms of mean SSIM with respect to the conventional CS at *AF* = 6. Also, the proposed technique shows 51.4%, 29.5%, 24.04%, and 18.0% improvements in terms of mean RMSE using the RMSProp, Adam, Adagrad, and Adadelta optimizers, respectively, with reference to the conventional CS at *AF* = 6. The results confirm that DWI U-Net performs better than the conventional CS reconstruction. Also, when comparing the different optimizers in DWI U-Net, RMSProp provides better results than the other optimizers.

## Introduction

Magnetic resonance imaging (MRI) is extensively used in medical imaging to produce inside images of the human body [1]. The key factor of MRI is excellent soft tissue contrast, better than other techniques in medical imaging [2], e.g., X-ray [3], computed tomography (CT) [4], and positron emission tomography (PET) [5]. Moreover, MRI is emerging as a promising noninvasive tool to assess organs such as the brain, kidney, and liver with the use of various sequences including T1 mapping and diffusion-weighted imaging (DWI) [1]. Long data acquisition time makes MRI challenging for some applications, e.g., dynamic imaging of the heart and abdomen, spinal imaging, and neuroimaging [6].

Diffusion-weighted magnetic resonance imaging (DWI-MR) introduces a new dimension to the analysis of MRI by adding functional details to anatomical images obtained by conventional sequences [7]. DWI utilizes the random and translational motion of water molecules, the so-called Brownian movement in biological tissues [8], to evaluate the molecular function and micro-architecture of the human body. Recently, DWI has been applied to detect the mechanism of extracellular diffusion of water molecules in biological tissues [9]. To generate a DWI-MR image, a readout signal is made dependent on applied diffusion gradients, which can be added to conventional MR sequences, e.g., a spin-echo sequence [10]. The most famous clinically used DWI-MR sequence is single-shot EPI (ss-EPI) [11].

The advantages of acquiring data along the ss-EPI trajectory [11] include fast coverage of *k*-space with a single RF pulse, but the long readout time of the EPI acquisition strategy leads to off-resonance effects, geometric distortion, and susceptibility artifacts in clinical settings [12]. To reduce these artifacts, under-sampled EPI data is acquired, but they lead to under-sampling artifacts [13].

Several reconstruction techniques have been proposed during the last decade to remove under-sampling artifacts, e.g., SENSE [14], GRAPPA [15], and Compressed Sensing (CS) [16]. However, these reconstruction techniques have some limitations, e.g., long reconstruction time, tuning of regularization parameters, and residual aliasing artifacts.

To accelerate the reconstruction time and to reduce the artifacts, faster reconstruction mechanisms may involve artificial intelligence [17], machine learning [18], and deep learning [19]. Deep neural networks (DNN) are now state-of-the-art machine learning models that are used in many fields, e.g., image recognition to natural language processing and computer vision [20] in both the industry and academia. The latest developments in DNN open new possibilities for an effective solution of the inverse problem of image reconstruction [21].

In the literature, neural networks have been used to model medical image reconstruction problems in CT and MRI [21, 22]. For image reconstruction, neural networks typically learn a proper transformation between the input (zero-filled under-sampled *k*-space) and the target (fully sampled *k*-space) by minimizing a specific loss function through a training process [21].

One popular neural network architecture for image denoising and reconstruction is U-Net [21]. Hu et al. [23] proposed a 2D U-Net network for ss-EPI distortion correction in the recent past. They used point-spread-function-encoded EPI (PSF-EPI) brain data as a reference to correct the traditional EPI distortion artifacts in neuroimaging.

Hu et al. [24] proposed accelerated multi-shot DWI-MR image reconstruction using deep learning for brain and breast DWI data with shot-to-shot phase correction. In this work, an unrolled pipeline containing recurrences of model-based gradient updates and neural networks was introduced. They combined MR physical model and U-Net in both *k*-space and image space as trainable priors. For in vivo brain and breast experiments, the network was trained initially on brain multi-shot DWI data and further fine-tuned for breast DWI data. The results presented in [24] showed that the proposed approach enabled almost real-time reconstruction for the brain and breast data with improved image quality, exhibiting the feasibility of multi-shot DWI in a wide range of clinical studies.

Bilgic et al. [25] recently proposed a reconstruction technique that uses a synergistic combination of machine learning and forward-model physics to demonstrate its implementation on structural and diffusion multi-shot EPI [25]. They utilized a patch-based U-Net network by splitting each shot of the multi-shot EPI into real and imaginary portions to get high-quality images with less distortion.

Kawamura et al. [26] used a technique proposed by Zhang et al. [27] for denoising DW images using a DNN to obtain high-resolution DW images based on multi-shot EPI. Kawamura et al. [26] performed 2D image denoising based on magnitude images only on each slice individually, not on all the slices as a whole.

Several studies have focused on the acceleration of MRI techniques through under-sampling and integration of a DNN into reconstruction [22]. Some researchers have focused on DWI reconstruction using a deep learning approach for denoising the ss-EPI [23] and multi-shot EPI acquisition strategies [25, 27, 28].

This paper proposes a U-Net-based reconstruction model (DWI U-Net) to reconstruct DWI-MR images from highly under**-**sampled 1D variable density Cartesian *k*-space data. The main objective of this work is to replace conventional reconstruction algorithms with deep learning–based reconstruction in DWI, which may help to reduce under-sampling artifacts, distortion artifacts, image reconstruction time, and computational burden of reconstructed images. The performance of the proposed method is compared with the conventional CS reconstruction [28]. Also, different optimizers (i.e., RMSProp, Adam, Adagrad, and Adadelta) are investigated to choose the best optimizer for the proposed DWI U-Net. The investigation of different optimizers for DWI U-Net is the real contribution of this work. Furthermore, the reconstruction results are compared with the state-of-the-art conventional CS reconstruction.

## Materials and Methods

This paper presents a DWI-MR image reconstruction approach using deep learning from highly under**-**sampled 1D variable density Cartesian *k*-space data.

### Proposed Method (DWI U-Net)

**Fig. 1:**
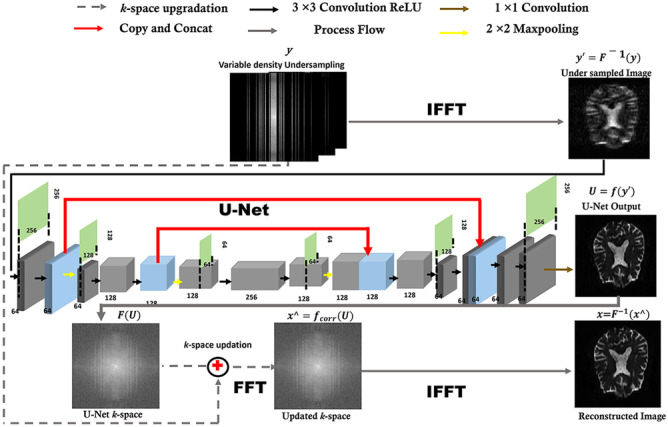
Schematic diagram of the proposed method (DWI U-Net), which comprises two main steps: (i) deep learning–based reconstruction using U-Net and (ii) *k*-space updation

Figure 1 shows a schematic diagram of the proposed method. Firstly, the variable density 1D Cartesian subsampling scheme is used to under-sample the *k*-space data $({\varvec{y}})$, where each slice is under-sampled differently to promote data sparsity and incoherent artifacts. The inverse Fourier transform of the under-sampled data $({{\varvec{y\acute{}}}={\varvec{F}}}^{-1}({\varvec{y}}))$ provides the aliased image ${\varvec{y\acute{}}}$**.** The ${\varvec{y\acute{}}}$ as an input and the artifact-free reference data as a label are fed to train U-Net. Once the network has been trained, the under-sampled unseen data are fed to the network to get the U-Net output (***U***), which recovers the zero-filled spaces of the under-sampled *k*-space data $({\varvec{y}})$. In doing so, it also distorts the originally acquired data points. To avoid this distortion and to retrospectively place the measured *k*-space data points in their corresponding original positions, an additional *k*-space updation step is applied, i.e., *k*-space correction ${\varvec{\hat{x}}}$= ***f***_**cor**_(***U***). After the *k*-space updation, the inverse Fourier transform (iFFT) is applied on the *k*-space (${\varvec{\hat{x}}}$) to obtain the solution image ${{\varvec{x}}={\varvec{F}}}^{-1\boldsymbol{ }}\left({\varvec{\hat{x}}}\right)$.

### Experimental Setup and Implementation

The proposed method (DWI U-Net) is trained and tested on open-source OASIS brain DWI datasets available at https://central.xnat.org/ [29] using Python 3.8. Human head data was acquired using a 3 T Siemens scanner, and the accompanying acquisition parameters were slice thickness = 2 mm, *TE* = 0.11 ms, *TR* = 14.5 ms, flip angle = ${90^\circ }$, and matrix size = 256 × 256. Data from thirteen healthy patients (i.e., 6 males and 7 females) with an age group of 41 ± 20 years is used in our experiments.

The *k*-space data from all the 13 patients were retrospectively under-sampled with variable density Cartesian under-sampling, and zero-filled images were produced using the inverse Fourier transform (iFFT). Each image was normalized linearly to have an intensity normalization between 0 and 1. The data of each patient contain images of different *b*-values, i.e., 0, 50, 100, 150, 200, 300, 350, 400, 450, 500, 600, 650, 700, and 800 s/mm^2^.

The proposed method is also tested on multidirectional DWI-MR human head data ($256\times 256\times 72\times 19$) obtained using a 3 T Siemens Prisma (Hospital University Geneva Switzerland) scanner. The data were acquired after the IRB/ethical approval committee. The accompanying acquisition parameters were slice thickness = 2 mm, *TE* = 54 ms, *TR* = 7400 ms, flip angle = ${90^\circ }$, matrix size = 256 × 256, and FOV = 230 × 230 mm. Multidirectional DWI-MR data contain images of different *b*-values, i.e., 0, 200, 400, and 1000 s/mm^2^.

The proposed U-Net architecture (DWI U-Net) used to reconstruct the DWI-MR image is shown in Fig. 1. The U-Net architecture contains both the encoding and decoding workflows. The size of the input and output data (image matrix size) is 256 × 256. In the proposed method, firstly, two $3 \times 3$ convolution layers are used each followed by rectified linear unit activation (ReLU) [30] to solve the vanishing gradient problem [21]. Convolution layers improve the efficiency of machine learning systems by extracting valuable features and hyperparameters, and introducing sparse interactions and equivariant representations of the input data [21, 31]. Secondly, we have applied a $2 \times 2$ max pooling operation with a stride of 2 for down-sampling; max pooling helps to make the representation roughly invariant to limited translations of the input [21, 31]. In the decoder path, upsampling instead of max pooling is used to restore the original size of the output. To get the desired size of the output image, upsampling of the feature channels followed by a $3 \times 3$ convolution layer and concatenation with the corresponding feature map from the contracting path is performed [32]. Finally, a 1 × 1 convolution is used in the last layer to combine each of the 64 features into one feature map to get the output.

To train and validate the proposed U-Net architecture, a training set having data from three patients’ whole brain volume images with a total of 6048 DWI images with 0 ≤ *b*-value ≤ 800 s/mm^2^ is used. The training set is decomposed into the training data with 5433 images and validation data of 605 images. The training set contains the under-sampled *k*-space data (input) and fully sampled images (labels). The trained network is tested on the OASIS data from 10 patients’ whole brain volume images having 20,160 testing set images. Furthermore, the trained network is tested on multidirectional data from one patient, i.e., whole brain volume having 1368 testing set images.

To train the proposed network, all the weights were initialized using a zero-centered normal distribution with a standard deviation of 0.01 without a bias term [21]. Optimization is one of the main components in deep learning, which makes the model training better during backpropagation when the weights are changed to minimize the loss error as well as fixes the “curse of dimensionality” problem [33].

In our work, mean square error is used as a loss function, which is minimized via the RMSProp, Adam, Adagrad, and Adadelta optimizers with a range of learning rates (1 × 10^−3^ to 1 × 10^−5^), mini-batch size = 5, epochs = 1000, and weight decaying factor 0.1. The proposed network training was implemented on Python 3.8 by Keras using TensorFlow as a backend on Intel(R), Xeon (R), CPU with 128 GB RAM, and GPU NVIDIA GeForce GTX 1080Tei,16 GB RAM using an early stopping criterion of 400 epochs in our experiments. The network required approximately 14 h for training in our experiments.

### Evaluation Parameters

The reconstructed image quality was assessed by measuring the mean structural similarity index measure (SSIM) [34], mean artifact power (AP) [35], mean root mean square error (RMSE) [34], and mean apparent diffusion coefficient (ADC) [36]. Furthermore, the proposed method and conventional CS results are statistically compared by the one-tailed Student *t*-test.

## Experimental Results

**Fig. 2:**
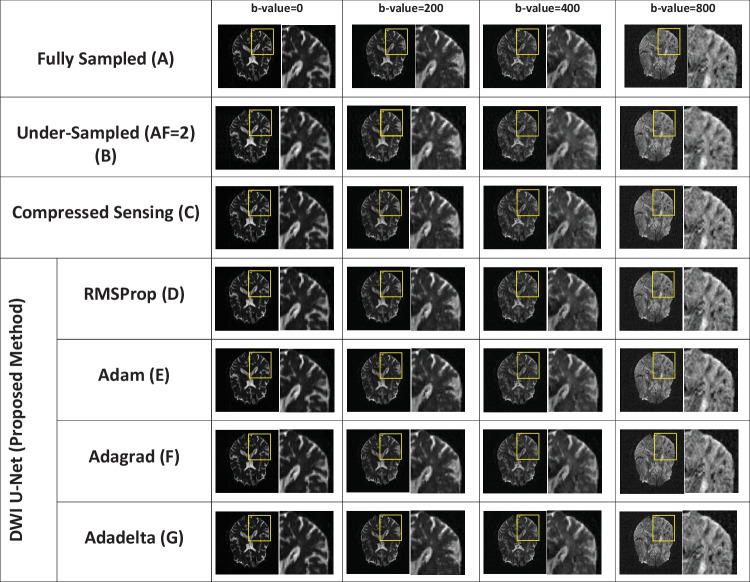
Reconstructed images of the human head OASIS dataset for the proposed method (DWI U-Net) with different optimizers, i.e., RMSProp, Adam, Adagrad, Adadelta, and Compressed Sensing at *AF* = 2 with different *b*-values, i.e., 0, 200, 400, and 800 s/mm^2^ (left to right in each column), respectively

**Fig. 3:**
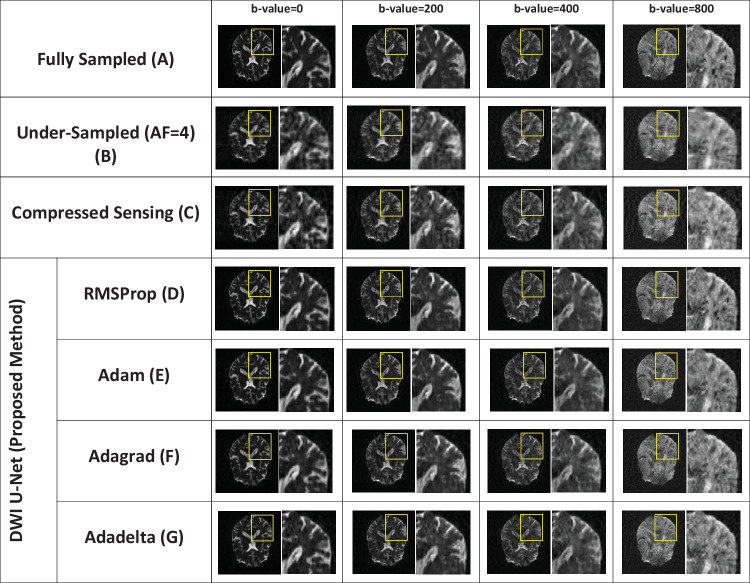
Reconstructed images of the human head OASIS dataset for the proposed method (DWI U-Net) with different optimizers, i.e., RMSProp, Adam, Adagrad, Adadelta, and Compressed Sensing at *AF* = 4 with different *b*-values, i.e., 0, 200, 400, and 800 s/mm^2^ (left to right in each column), respectively

**Fig. 4:**
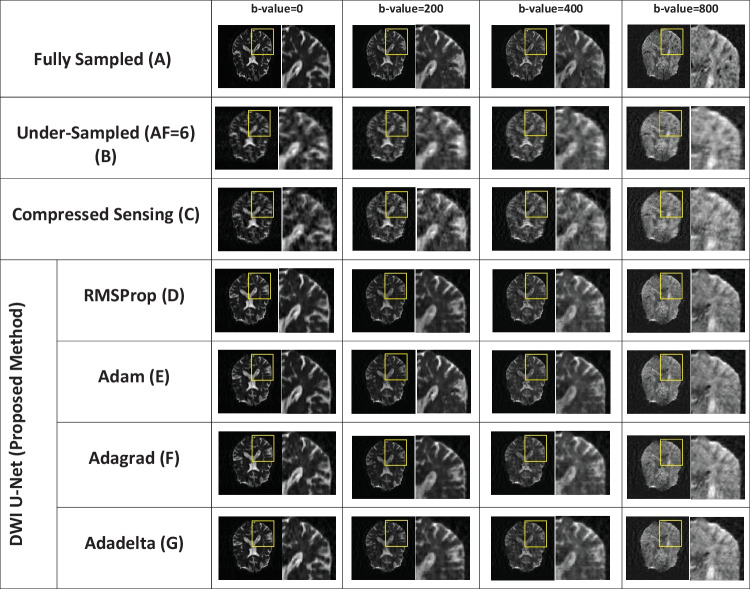
Reconstructed images of the human head OASIS dataset for the proposed method (DWI U-Net) with different optimizers, i.e., RMSProp, Adam, Adagrad, Adadelta, and Compressed Sensing at *AF* = 6 with different *b*-values, i.e., 0, 200, 400, and 800 s/mm^2^ (left to right in each column), respectively

**Fig. 5:**
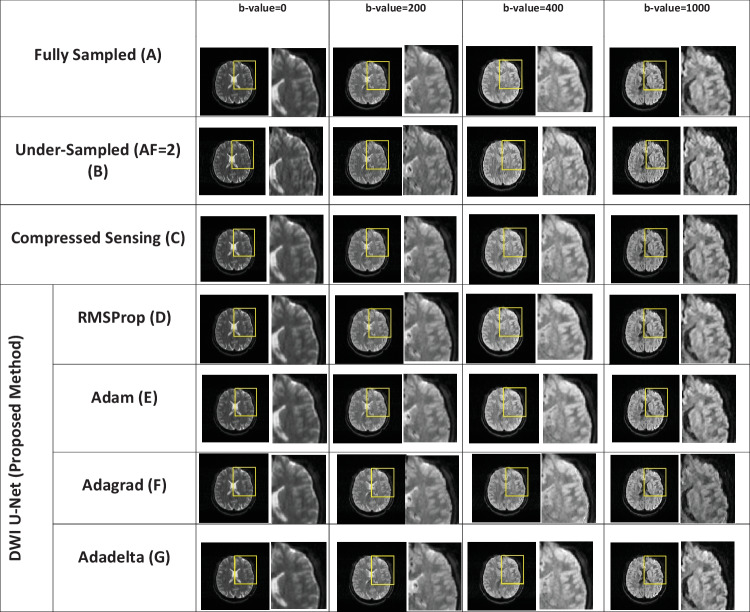
Reconstructed images of the human head multidirectional dataset for the proposed method (DWI U-Net) with different optimizers, i.e., RMSProp, Adam, Adagrad, Adadelta, and Compressed Sensing at *AF* = 2 with different *b*-values, i.e., 0, 200, 400, and 1000 s/mm.^2^ (left to right in each column)

**Fig. 6:**
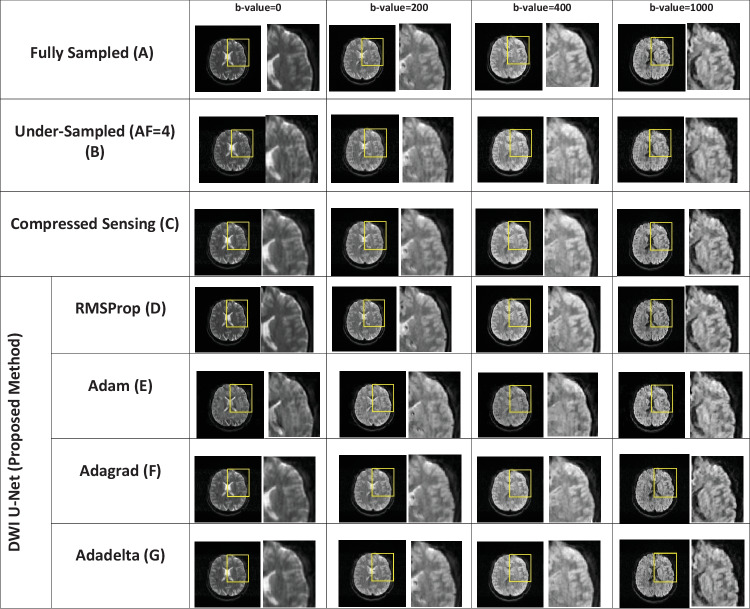
Reconstructed images of the human head multidirectional dataset for the proposed method (DWI U-Net) with different optimizers, i.e., RMSProp, Adam, Adagrad, Adadelta, and Compressed Sensing at *AF* = 4 with different *b*-values, i.e., 0, 200, 400, and 1000 s/mm.^2^ (left to right in each column)

**Fig. 7:**
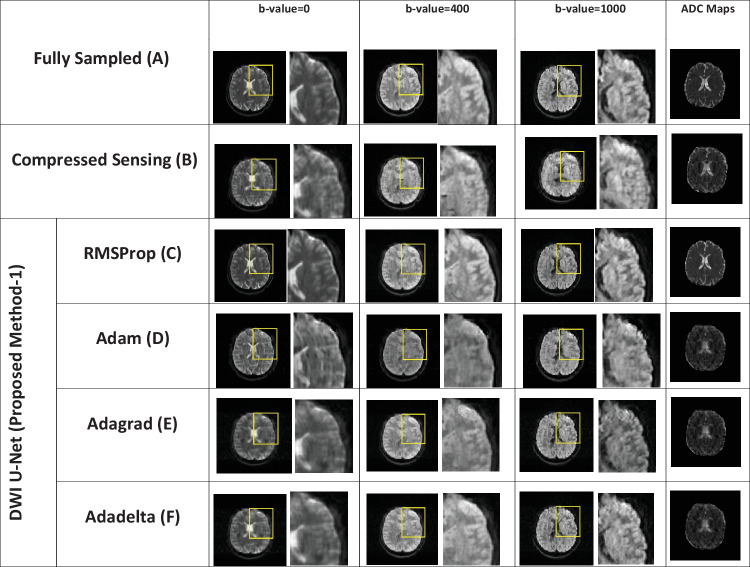
Reconstructed images of the human head multidirectional dataset for the proposed method (DWI U-Net) with different optimizers, i.e., RMSProp, Adam, Adagrad, Adadelta, and Compressed Sensing at *AF* = 6 with different *b*-values, i.e., 0, 200, 400, and 1000 s/mm.^2^ (left to right in each column)

The DWI image reconstruction is performed for the whole brain volume with different acceleration factors, i.e., *AF* = 2, 4, and 6, and different *b*-values, i.e., 0, 50, 100, 150, 200, 300, 350, 400, 450, 500, 600, 650, 700, 800, and 1000 s/mm^2^. For simplicity, the central slice of reconstructed images with *b*-values of 0, 200, 400, and 800 s/mm^2^ is shown in Figs. 2, 3, and 4. For further visual assessment, the reconstructed slices of three patients from the OASIS brain DWI dataset are also given in the supporting documents (see Appendix). Furthermore, the central slice of an empirically chosen multidirectional dataset with *b*-values of 0, 200, 400, and 1000 s/mm^2^ is shown in Figs. 5, 6, and 7.

Figures 2, 3, 4, 5, 6, and 7 show the reconstructed images of the two different datasets (i.e., OASIS dataset and multidirectional dataset) using the proposed DWI U-Net with different optimizers and conventional CS [28]. In each figure, row A shows the fully sampled data, row B shows the under-sampled data, row C shows the reconstruction results of the conventional Compressed Sensing [28], row D shows the reconstruction results of DWI U-Net with the RMSProp optimizer, row E shows the reconstruction results of DWI U-Net with the Adam optimizer, row F shows the reconstruction results of DWI U-Net with the Adagrad optimizer, and row G shows the reconstruction results of DWI U-Net with the Adadelta optimizer. All the data are simulated at *AF* = 2, 4, and 6 and different *b*-values ranging from 0 ≤ *b*-values ≤ 1000 s/mm^2^. In Figs. 2, 3, 4, 5, 6, and 7, the *b*-value changes from left to right for each *AF*. For enhanced visualization of the reconstruction quality, a magnified region of each image is displayed.

Figures 2, 3, and 4 show the results of the OASIS dataset using the proposed DWI U-Net with different optimizers and conventional CS. In Figs. 2, 3, and 4, the results show that the proposed method efficiently reconstructs the solution image at *AF* = 2, 4, and 6 while the conventional CS leaves some artifacts. Furthermore, the RMSProp optimizer provides better results than the other optimizers in DWI U-Net. The results from the Adam, Adagrad, and Adadelta optimizers contain comparatively greater blurring and artifacts in the reconstructed images than the RMSProp optimizer. The RMSProp in DWI U-Net reconstructs good-quality results at lower as well as higher *b*-values than the other optimizers. This might be because RMSProp splits the learning rate by an exponential decaying average of the squared gradient [37, 38].

Table 1 shows the results in terms of “mean ± std” of AP, RMSE, and SSIM values with the proposed method (DWI U-Net) for different optimizers, i.e., RMSProp, Adam, Adagrad, and Adadelta, and conventional compressed sensing [28] for OASIS dataset brain DWI central slice data at *AF* = 2, 4, and 6.

**Table 1:** Comparison of the reconstruction quality in terms of “mean ± std” of AP, RMSE, and SSIM values for the human head OASIS dataset at acceleration factors of 2, 4, and 6 between the proposed DWI U-Net with different optimizers, i.e., RMSprop, Adam, Adagrad, Adadelta, and conventional Compressed Sensing at *p* < 0.05

**Methods**		**AF = 2**			**AF = 4**			**AF = 6**		
		***Mean AP* ± *STD***	***Mean RMSE* ± *STD***	***Mean SSIM* ± *STD***	***Mean AP* ± *STD***	***Mean RMSE* ± *STD***	***Mean SSIM* ± *STD***	***Mean AP* ± *STD***	***Mean RMSE* ± *STD***	***Mean SSIM* ± *STD***
Proposed Method (DWI U-Net)	RMSProp	0.0010 ± 0.0003	0.0035 ± 0.0005	0.9950 ± 0.290	0.0101 ± 0.0020	0.0171 ± 0.0023	0.9656 ± 0.0310	0.0140 ± 0.0026	0.0204 ± 0.0013	0.9554 ± 0.0530
	Adam	0.0012 ± 0.0005	0.0040 ± 0.0006	0.9696 ± 0.0202	0.0119 ± 0.0050	0.0185 ± 0.0050	0.9480 ± 0.0230	0.0144 ± 0.0035	0.0296 ± 0.0041	0.9219 ± 0.0223
	Adagrad	0.0021 ± 0.0006	0.0055 ± 0.0012	0.9624 ± 0.0197	0.0175 ± 0.0060	0.0227 ± 0.0082	0.9431 ± 0.0210	0.0249 ± 0.0074	0.0319 ± 0.0050	0.9145 ± 0.0220
	Adadelta	0.0026 ± 0.0008	0.0064 ± 0.0015	0.9619 ± 0.0143	0.0184 ± 0.0065	0.0234 ± 0.0090	0.9408 ± 0.0190	0.0255 ± 0.0075	0.0344 ± 0.0058	0.9142 ± 0.0200
Conventional Method	Compressed Sensing	0.0029 ± 0.0013	0.0071 ± 0.0010	0.9292 ± 0.0017	0.0191 ± 0.0089	0.0300 ± 0.0099	0.8856 ± 0.0100	0.0358 ± 0.0080	0.0420 ± 0.0087	0.8407 ± 0.0120

At *AF* = 6, the proposed technique provides 61.1%, 60.0%, 30.4%, and 28.7% improvements in terms of mean AP as compared to the conventional CS for DWI U-Net with the RMSProp, Adam, Adagrad, and Adadelta optimizers, respectively. Furthermore, the results of DWI U-Net with the RMSProp, Adam, Adagrad, and Adadelta optimizers in terms of mean RMSE values show an improvement of 51.4%, 29.5%, 24.04%, and 18.0% with respect to the conventional CS at *AF* = 6. Also, the proposed technique shows 13.6%, 10.0%, 8.7%, and 8.74% improvements in terms of mean SSIM values using the different optimizers, i.e., RMSProp, Adam, Adagrad, and Adadelta, respectively, with reference to the conventional CS at *AF* = 6.

Furthermore, the results show that the RMSProp in DWI U-Net provides lower AP and RMSE values and higher SSIM values as compared to the other optimizers. The results show a significant improvement in image quality with the proposed DWI U-Net than the conventional CS in terms of AP, RMSE, and SSIM values in our experiments.

In Figs. 5, 6, and 7, the reconstruction results of the human head multidirectional DWI data are shown at *AF* = 2, 4, and 6 with different *b*-values, i.e., 0, 200, 400, and 1000 s/mm^2^. The results show that the proposed method efficiently reconstructs the solution image while the conventional CS leaves some artifacts in the reconstructed images. Furthermore, the results confirm that the proposed DWI U-Net with the RMSProp optimizer recovers the solution image better than other optimizers.

Table 2 shows the results of multidirectional brain DWI data central slices in terms of “mean ± std” of AP, RMSE, and SSIM values at *p* < 0.05 for the proposed method (DWI U-Net) with different optimizers and conventional CS [28] at *AF* = 2, 4, and 6.

**Table 2:** Comparison of the reconstruction quality in terms of “mean ± std” of AP, RMSE, and SSIM values for the human head multidirectional dataset at acceleration factors of 2, 4, and 6 between the proposed method (DWI U-Net) having different optimizers, i.e., RMSprop, Adam, Adagrad, Adadelta, and conventional Compressed Sensing at *p* < 0.05

**Methods**		**AF = 2**			**AF = 4**			**AF = **6		
		***Mean*** ***AP* ± *STD***	***Mean RMSE* ± *STD***	***Mean SSIM* ± *STD***	***Mean AP* ± *STD***	***Mean RMSE* ± *STD***	***Mean SSIM* ± *STD***	***Mean AP* ± *STD***	***Mean RMSE* ± *STD***	***Mean SSIM* ± *STD***
Proposed Method (DWI U-Net)	RMSProp	0.0043 ± 0.0032	0.0085 ± 0.0017	0.9933 ± 0.0356	0.0079 ± 0.0046	0.0150 ± 0.0036	0.9693 ± 0.0423	0.0202 ± 0.0066	0.0255 ± 0.0062	0.9586 ± 0.0485
	Adam	0.0058 ± 0.0038	0.0096 ± 0.0028	0.9450 ± 0.0256	0.0096 ± 0.0049	0.0175 ± 0.0044	0.9393 ± 0.0294	0.0228 ± 0.0085	0.0331 ± 0.0080	0.9188 ± 0.0350
	Adagrad	0.0072 ± 0.0039	0.01040.0029	0.9446 ± 0.0236	0.0164 ± 0.0057	0.0236 ± 0.0053	0.9194 ± 0.0286	0.0290 ± 0.0092	0.0355 ± 0.0087	0.9212 ± 0.0302
	Adadelta	0.0079 ± 0.0040	0.0109 ± 0.0030	0.9382 ± 0.0209	0.0169 ± 0.0064	0.0242 ± 0.0059	0.9139 ± 0.0256	0.0315 ± 0.0099	0.0389 ± 0.0097	0.9154 ± 0.0294
Conventional Method	Compressed Sensing	0.0101 ± 0.0070	0.0145 ± 0.0060	0.8911 ± 0.0056	0.0233 ± 0.0102	0.0281 ± 0.0079	0.8511 ± 0.0146	0.0371 ± 0.0150	0.0416 ± 0.0145	0.8123 ± 0.0180

At a higher acceleration factor, i.e., *AF* = 6, the proposed method provides an improvement of 45.5%, 38.5%, 21.8%, and 15.0% in terms of mean AP for DWI U-Net with the different optimizers, i.e., RMSProp, Adam, Adagrad, and Adadelta, respectively, as compared to the conventional CS. Similarly, the results of DWI U-Net with the RMSProp, Adam, Adagrad, and Adadelta optimizers in terms of mean RMSE show an improvement of 38.7%, 20.4%, 14.6%, and 6.4%, respectively, with reference to the conventional CS at *AF* = 6. Also, the proposed technique shows 18.0%, 13.1%, 13.4%, and 12.5% improvement in terms of mean SSIM with the RMSProp, Adam, Adagrad, and Adadelta optimizers, respectively, as compared to the conventional CS at *AF* = 6.

The RMSProp optimizer in DWI U-Net provides lower AP and RMSE values and higher SSIM values as compared to the other optimizers in DWI U-Net. The results confirm that significant improvements in terms of evaluation parameters (i.e., AP, RMSE, and SSIM) have been obtained with the proposed method (DWI U-Net) than the conventional CS.

**Fig. 8:**
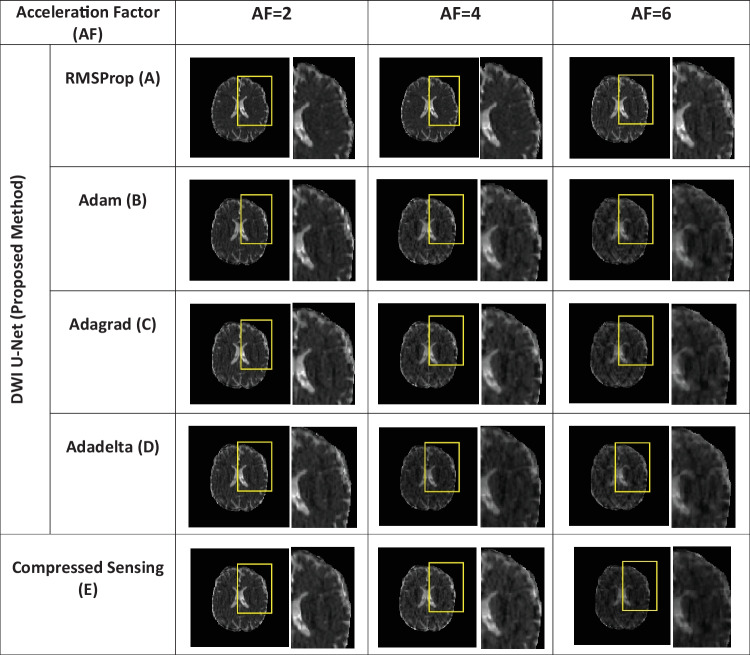
Mean ADC maps of the multidirectional human head dataset for the proposed method (DWI U-Net) with different optimizers, i.e., RMSProp, Adam, Adagrad, Adadelta, and Ccompressed Sensing at *AF* = 2, 4, and 6 (top to bottom in row)

Figure 8 shows the mean apparent diffusion coefficient (ADC) maps of multidirectional data using the proposed DWI U-Net with different optimizers and conventional CS at *AF* = 2, 4, and 6. In Fig. 8, row A shows the mean ADC map of DWI U-Net with the RMSProp optimizer, row B shows the mean ADC map of DWI U-Net with the Adam optimizer, row C shows the mean ADC map of DWI U-Net with the Adagrad optimizer, row D shows the mean ADC map of DWI U-Net with the Adadelta optimizer, and row E shows the mean ADC map of the conventional compressed sensing. The ADC maps show that the RMSProp in DWI U-Net provides more visible corpus callosum, white matter, and grey matter, and less blurring artifacts as compared to the other optimizers and conventional CS.

**Fig. 9:**
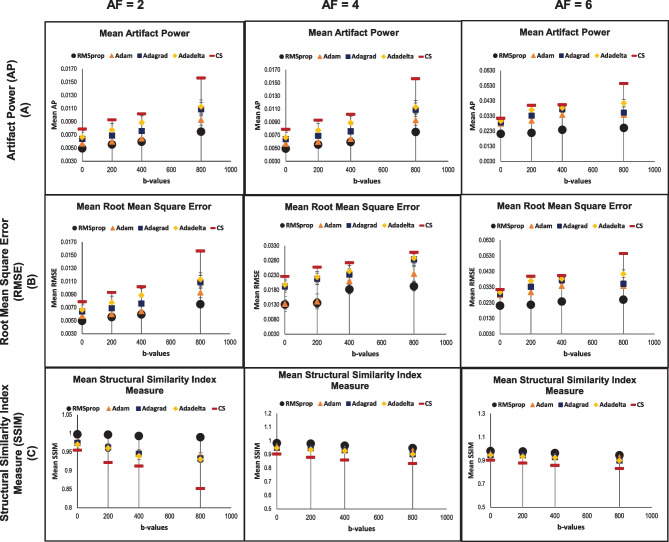
Comparison of the reconstruction quality on the basis of results for the data obtained from 10 patients (OASIS dataset) in terms of mean evaluation parameters, i.e., AP (row A), RMSE (row B), and SSIM (row C) for 3 T brain DWI data at (2 ≤ *AF* ≤ 6) between DWI U-Net having different optimizers, i.e., RMSprop, Adam, Adagrad, and Adadelta, and conventional Compressed Sensing (*p* < 0.05)

Figure 9 shows the overall performance trends of the evaluation parameters for DWI U-Net with different optimizers and conventional CS for all the10 patients (OASIS dataset) with different *b*-values, i.e., 0, 200, 400, and 800 s/mm^2^ at *AF* = 2, 4, and 6. These plots confirm that the RMSProp optimizer provides lower AP and RMSE values and higher SSIM than the other optimizers in DWI U-Net as well as better results as compared to the conventional CS for all the *AF*s and *b*-values. Therefore, we can conclude that DWI U-Net with the RMSProp optimizer provides better results in terms of mean AP, mean RMSE, and mean SSIM parameters as compared to the other optimizers in DWI U-Net (*p* < 0.05). Also, it has been observed that DWI U-Net provides overall better results than the conventional CS for all the *AF*s and *b*-values.

## Discussion

Diffusion-weighted imaging (DWI) has revolutionized MRI [7, 39]. ss-EPI is the most clinically used DWI sequence but suffers from off-resonance effects, geometric distortion, and susceptibility artifacts due to faster switching of DWI gradients resulting in low SNR in the final image [12]. DWI artifacts can be avoided by acquiring lesser amount of data (under-sampled acquisition against Nyquist criterion), but it leads to under-sampling artifacts [13]. This paper proposes a deep learning–based method, i.e., DWI U-Net, for 1D variable density under-sampled data to get artifact-free DW images. Furthermore, in this paper, a comparison of different optimizers for DWI data with U-Net has also been performed, and the results are compared with conventional CS reconstruction.

Experiments are performed on the whole brain volume OASIS DWI-MR dataset of 13 healthy volunteers at various acceleration factors (2 ≤ *AF* ≤ 6) acquired with different *b*-values, i.e., 0, 200, 400, and 800 s/mm^2^. Also, the proposed method is tested on multidirectional DWI-MR human head data acquired with different *b*-values 0, 200, 400, and 1000 s/mm^2^. The experimental results shown in Figs. 2, 3, 4, 5, 6, and 7 confirm that the proposed method successfully reconstructs the solution images for different *b*-values, i.e., 0, 200, 400, 800, and 1000 s/mm^2^ with *AF* = 2, 4, and 6. The reconstruction results for the different datasets (i.e., OASIS dataset and multidirectional dataset) with respect to “mean ± std” of AP, RMSE, and SSIM values are given in Tables 1 and 2 to compare the reconstruction quality of the different optimizers and conventional CS at *p* < 0.05.

Evident from the evaluation parameters and visual assessment of the reconstruction results, the proposed scheme provides better results in terms of AP, RMSE, and SSIM values at different acceleration factors, i.e., 2, 4, and 6, for different *b*-values.

In DWI, images at lower *b*-values contain less diffusion information and less artifacts than those at higher *b*-values [40]. All the optimizers in DWI U-Net show promising reconstruction results at a lower *AF* and low *b*-values. Furthermore, the RMSProp in DWI U-Net shows better results than the other optimizers as well as the conventional CS. This may be because RMSProp well divides the learning rate by an exponentially decaying average of a squared gradient [37] that helps to converge quickly [41]. Similarly, at a lower acceleration factor and higher *b*-values, e.g., *AF* = 2 with *b*-value = 1000 s/mm^2^, DWI U-Net RMSProp gives good-quality results with less artifacts, whereas the other optimizers in DWI U-Net provide more artifacts as shown in Fig. 5. Also, at a higher acceleration factor and lower *b-*values, the RMSProp optimizer provides better results as compared to the other optimizers in DWI U-Net as well as the conventional CS as shown in Figs. 4 and 7.

In this study, we investigated deep learning–based reconstruction via different optimizers and CS for the whole brain volume at different acceleration factors, i.e., 2 ≤ *AF* ≤ 6, with *b*-values ranging between 0 ≤ *b*-values ≤ 1000 s/mm^2^. Here, we discuss failure cases based on the percentage of images that failed to reconstruct for Adam, Adagrad, and Adadelta based U-Net and CS with reference to RMSprop U-Net (proposed method). The Adam optimizer failed to recover 12% of all the images as compared to RMSprop U-Net. Similarly, Adagrad, Adadelta, and CS failed to recover 22%, 27%, and 37% of all the images with reference to RMSprop U-Net at *AF* = 2. At *AF* = 4, Adam Adagrad, Adadelta, and CS failed to recover 22%, 23%, 25 and 34% of all the images, respectively, as compared to RMSprop U-Net. At a higher *AF* (i.e., = 6 in this paper), Adam, Adagrad, Adadelta, and CS failed to reconstruct 16%, 25%, 28%, and 40% of all the images as compared to the proposed RMSprop U-Net. In our experiments, RMSprop performs better than the other optimizers as well as CS for both the lower and higher *b*-values as well as for *AF* = 2, 4, and 6.

The proposed method successfully removes under-sampling artifacts at both lower *b*-values and higher *b*-values, while CS does not perform well at higher *b*-values. This is because the high *b*-values contain more diffusion information and strong background signal suppression. The assessment parameters demonstrate that the proposed method noticeably removes artifacts and gives good reconstruction results even at higher *b*-values. As compared to our proposed method, CS fails to give good reconstruction results at higher *b*-values as the DWI contrast decreases with an increase in *b*-value due to an increased diffusion gradient strength. As a result, the features with low contrast are submerged by the interference and not recovered by CS [16]. However, the proposed DWI U-Net learns the features of lower as well as higher *b*-values during network training. Hence, the proposed method performs better as compared to the conventional CS at all the *b*-values.

We used ReLU as an activation function, and one of the main reasons for using ReLU [42] is that it does not activate all neurons at the same time in a neural network that makes the proposed DWI U-Net less computationally expensive [30].

To summarize the above discussion, the reconstruction results of our experiments with DWI U-Net using the RMSprop, Adam, Adagrad, and Adadelta optimizers, and conventional Compressed Sensing show that DWI U-Net (with the RMSProp optimizer) provides better results. The images reconstructed with the RMSProp in DWI U-Net are close to the fully sampled images at all the *b*-values. In the future, the proposed method can be expanded to multichannel data, with appropriate variations in the sampling pattern and learning network.

## Conclusion

The present study proposes a deep learning–based DWI U-Net for DWI image reconstruction. The proposed method is tested on the whole brain volume at different acceleration factors, i.e., 2 ≤ *AF* ≤ 6, with *b*-values ranging between 0 ≤ *b*-values ≤ 1000 s/mm^2^. The proposed method presents substantially improved results as compared to conventional CS reconstruction in terms of quality assessment parameters, i.e., mean AP, mean RMSE, mean SSIM, and mean ADC at *AF* = 2, 4, and 6. Also, the results confirm that the proposed DWI U-Net with the RMSProp optimizer recovers better quality images than with the other optimizers, i.e., Adam, Adagrad, and Adadelta.
